# Acuity, Diagnosis, and Community Factors in Virtual and In-Person Treatment: Qualitative Study

**DOI:** 10.2196/82863

**Published:** 2026-04-20

**Authors:** Brianna Cerrito, Alexa Connors, Amanda Fialk, Jamie Xiao, Josephine Usow, Frank D Buono

**Affiliations:** 1 Research & Innovation Department, The Dorm, New York, NY, United States; 2 Clinical Department, The Dorm, New York, NY, United States; 3 Department of Psychiatry, Yale School of Medicine, 300 George Street, New Haven, CT, 06511, United States, 1 203 292 0980

**Keywords:** face-to-face, online group psychotherapy, online treatment, qualitative methods, therapeutic relationship, video-delivered psychotherapy, acuity, diagnosis, community

## Abstract

**Background:**

The COVID-19 pandemic accelerated the adoption of virtual psychotherapy, making videoconferencing a vital tool for maintaining continuity of care. While virtual sessions offer flexibility and accessibility, concern has also been raised about the impact of virtual care on therapeutic relationships, a sense of community, and the ability to support clients with higher levels of clinical need.

**Objective:**

This study sought to examine how mediums of care (ie, virtual vs in-person) impact the therapeutic experience for clients and providers in critical domains such as community, clinical presentation, and clinicians’ use of clinical skills. The following themes were evaluated: (1) How therapeutic relationships and community dynamics differ across virtual and in-person mediums of mental health care? (2) How acuity and diagnosis influence the perception of effectiveness and safety of these mediums?

**Methods:**

This study is a grounded theory design. Participants were intensive outpatient mental health clients and clinicians. Participants were interviewed in focus groups of either 6‐8 clinicians or clients, assessing for important themes (ie, attendance, engagement, and therapeutic alliance) across the 2 modes of care (ie, virtual vs in-person). A total of 6 structured focus groups were conducted. Thematic analysis was conducted to analyze the transcripts until theoretical saturation was achieved.

**Results:**

Results highlighted the nuanced differences in client and clinician perceptions of relational dynamics and effectiveness of care in virtual versus in-person mental health care. This study revealed, the perspectives of the client and clinician, that in-person treatment fosters more engagement and interpersonal connection (ie, therapeutic alliance and peer-to-peer) than virtual settings. Additionally, in-person care better supports the ability to exercise important clinical skills, especially for highly acuity clients with complex diagnoses.

**Conclusions:**

The results of this study outline how the medium of care (ie, virtual vs in-person) impacts therapeutic experience from the perspective of both client and clinician. It is understood that critical domains such as community, interpersonal relationships, and clinicians’ use of clinical skills are affected by the medium of care, and key factors such as clinical presentation (ie, acuity and diagnosis) must be accounted for when choosing a particular mode of care for a client.

## Introduction

Virtual psychotherapy appointments saw an increase during the COVID-19 pandemic, helping to maintain continuity of care without violating social guidelines [1]. Years after the pandemic, virtual psychotherapy is still relevant for many populations, including those in rural areas, of marginalized communities, and with demanding careers, offering flexibility, convenience, and access to care for clients and providers [2,3]. In-home therapy sessions have increased, and some clients experience greater openness and relaxation in therapy, allowing for deeper conversations about their mental health struggles [4]. However, virtual sessions also present additional challenges to therapy, such as disrupting the ability to read body language and pick up on nonverbal cues. For individuals who struggle with activities of daily living (ADLs), a therapist may not be able to thoroughly assess whether a client has showered or brushed their teeth, due to the lack of physical presence [5]. Other challenges to virtual therapy include concerns about establishing strong therapeutic relationships via online mediums, as well as the ability to care for clients with higher clinical acuity, as these clients may need a more involved approach [6-8].

In therapy, human connection (ie, therapeutic alliance and community-building) is crucial to positive mental health outcomes (ie, depression, anxiety, stress, and suicidal ideation and attempts) [9,10]. Therapeutic alliance is a foundation of psychotherapy, representing the trust and effort maintained by both parties to increase mental well-being [11]. The link between therapeutic alliance and positive outcomes is critical, where the therapeutic relationship acts as a mediator of change in mental health outcomes [12]. Research has also consistently demonstrated the importance of having strong relationships with others, reporting that a sense of community and higher levels of social support are positively correlated with reduced mental health symptoms and improved well-being [10,13]. Social relationships both within and outside treatment spaces are a critical part of the therapeutic process, and the changed nature of relationships over virtual mediums raises new questions into the quality of online relationships, and whether these online relationships may have an impact on mental health outcomes.

Research conducted during and since the COVID-19 pandemic highlights differing perspectives on the nature of virtual relationships in therapeutic settings. Some research has shown that clients are able to create a strong connection in virtual mental health care settings, with reports of no change in the quality of therapeutic relationship [1,14,15]. However, systematic reviews and meta-analyses following the COVID-19 pandemic suggest that teletherapy may hinder therapeutic alliance, as it has been shown to foster a weaker therapeutic alliance when compared to face-to-face settings [16,17]. Other research reports therapists’ perceptions of virtual mediums as less effective due to the barrier of a screen and reduced authenticity when providing care [6,18]. The varied responses call for further research on differences between the perceived quality of in-person and virtual therapeutic relationships.

Virtual mediums may also impact a therapist’s ability to appropriately treat high acuity clients. In virtual settings, therapists have reported a decreased ability to practice clinical skills and respond in crisis or emergency, which is especially important for high acuity clients, as they need more robust care [8,19]. Research has also shown that clients with higher baseline symptom severity demonstrate greater mental health outcomes as well as shorter length of stay in face-to-face versus remote treatment settings, suggesting that there may be a potential benefit to in-person care for high acuity clients [7,19].

While existing literature emphasizes the importance of in-person connection for therapeutic quality, there is a gap in existing literature on how clinicians and clients perceive key therapeutic factors, such as client acuity, diagnosis, and engagement, within virtual versus in-person treatment contexts. Notably, much of the existing teletherapy literature focuses on symptom outcomes or therapeutic alliance broadly, with limited qualitative examination of how clinicians and clients jointly experience acuity, safety, and clinical skill use across care modalities, particularly in higher acuity settings. This study explored how mediums of care (ie, virtual vs in-person) impact the therapeutic experience for young adult clients and providers in an intensive outpatient mental health setting across critical domains such as community, clinical presentation, and clinicians’ use of clinical skills. Given there are benefits and drawbacks across virtual and in-person mediums of care, this paper explored 2 main themes from the perspective of clinicians and their clients. The research questions evaluated the ways in which therapeutic relationships and community dynamics differ across virtual and in-person mediums of mental health care and how acuity and diagnosis influence the perception of effectiveness and safety of these mediums.

## Methods

### Study Design

This study uses a grounded theory design, a qualitative research method aimed at developing a theory based on the data collected [20]. Initially, grounded theory methods were used to arrange and code the data, allowing the development of tentative categories. Reflexive thematic analysis, rooted in constructivist principles, then facilitated the identification and analysis of patterns across the data [20]. Through iterative coding and constant comparison, grounded theory methods helped in refining these patterns into themes, ultimately leading to an explanatory framework that elucidates the relationships among identified themes. The present researchers sought to develop a theory directly based on the data collected to understand the participants’ experiences. The research questions were as follows: How do therapeutic relationships and community dynamics differ across virtual and in-person care? How does acuity of mental health diagnoses influence these perceptions?

### Participants

Participants included clinical staff (ie, those with social work degrees, professional counseling degrees, nursing degrees, Master of Arts degrees, mental health counseling degrees, alcohol and substance use degrees, and dietetic degrees) and clients seeking intensive outpatient mental health treatment in New York, NY, and Washington, DC. The study included 32 individuals (14 clients and 18 clinical staff members) broken into 6 homogenous groups, 3 of which were client groups and 3 of which were staff groups.

Groups did not exceed 4 to 6 individuals, and this was intentional to provide ample opportunity for engagement of each respondent in the groups. During the focus groups, the client respondents were in 1 of 2 locations: home or a mental health treatment center. However, all clinicians were on site at the treatment program for focus group interviews.

Client demographics, including age, race or ethnicity, and gender identity, and clinical credentials for staff were consistent with those previously reported in Cerrito et al [5]. From the point of view of the analyses conducted, the distribution of the sociodemographic factors remains important, as the sample was predominantly White (n=12, N=14, 86%), and majority were cisgender male and female (n=11, N=14, 79%), with a smaller portion identifying as transgender nonbinary (n=3, N=14, 21%), which may limit generalizability.

### Ethical Considerations

This study is approved by the institutional review board at Yale School of Medicine (IRB #2000032626). Clinical staff and clients who had sought intensive outpatient mental health treatment since the pandemic provided insight about their experience through qualitative focus groups. All participants provided informed consent prior to participation. Data were deidentified and stored securely to protect participant privacy and confidentiality. No financial compensation was provided for participation.

### Data Collection

This research followed the methodology outlined in Cerrito et al [5]. Recruitment occurred via an email invitation outlining the study’s aims, the voluntary nature of participation, and the opportunity to contribute to advancing understanding of mediums of mental health care. Data collection occurred between March 20 and April 3, 2023, with participation invitations sent via email to staff and clients. Inclusion criteria were as follows: (1) at least 18 years of age, (2) are a client previously admitted to The Dorm or are a provider employed by The Dorm, and (3) able to read and understand the consent form. Exclusion criteria included the following: (1) are unfit to complete the survey due to medical or psychological constraints and (2) require a legally authorized representative.

In qualitative research, the social context of the participants’ lives, time, place, and biography are important [21]. Client participant perspectives were interpreted within a shared social and clinical context, including engagement in intensive outpatient mental health care during the pre- and postpandemic period, while clinical staff participants had experience delivering both in-person and virtual treatment modalities. This was also the subject of attention both during the interview and the analytical process.

The invitation to participate in this study was sent out in emails that included detailed descriptions of the subject of the study, its course, and the purpose of the data being solely for scientific purposes. All the respondents gave informed verbal consent to participate in the study at the start of the focus groups. To minimize interviewer preconceptions, focus groups were conducted by a qualitative researcher trained in neutral, open-ended interviewing techniques that use nonleading prompts. These prompts were consistent with Braun and Clarke’s reflexive thematic analysis approach, which emphasizes reflexivity, openness to participant meaning, and minimizing assumptions [22].

The first part of the interview guide was related to attendance and engagement in the 2 modes of care, and the second portion began to explore detailed topics, referring to a list of auxiliary questions, which had a semistructured form. To obtain the best quality of data possible, the researcher asked detailed questions about experiences and reflections related to engagement, friendships, rapport-building, trust, honesty, communication, and the benefits of virtual versus in-person. The questions were open and nonleading, and follow-up items were prewritten to ensure comprehensive dialogue, for example, “Tell me about attendance in virtual care settings.”

During the interview, all respondents were consistently encouraged to share their experiences as openly and honestly as possible. The interviewer used nonjudgmental language and flexible pacing to ensure that all respondents had a chance to contribute. Much effort was made to create optimal comfort and safety in the space for this purpose.

### Data Analysis

Throughout the data analysis process, the interpretative perspectives of clients and clinical staff were prioritized, consistent with grounded theory methodological assumptions. The analysis began with grounded theory techniques like open coding and constant comparison, which helped in the initial organization of the data [23]. Reflexive thematic analysis was then used to further analyze and report patterns, guided by Braun and Clarke’s 6-phase framework [21]. This structured approach included familiarization with data, generating initial codes, and identifying themes [21,22]. Constant comparison was used to refine these themes into conceptual categories, contributing to the development of theoretical insights into the impacts of different care mediums [21]. For instance, themes such as client engagement, friendship, and rapport were identified as central to the sense of community. Through constant comparison, these themes were linked to broader constructs like therapeutic alliance and community dynamics, leading to theoretical insights into the differential effects of virtual and in-person care on these constructs.

The authors remained open to the depth of the transcripts and were responsive to emerging insights throughout the analytic process. The stages of this research study were adapted from Braun and Clarke’s 6-phase thematic analysis framework, which include familiarization with data, generating initial codes, searching for themes, reviewing themes, and defining and naming themes, and the final phase 6, writing the report [22].

Thematic analysis in this study went in the following stages.

- Stage 1: All 3 transcripts were reviewed by each of the 3 authors (BC, AC, and AF) to uncover central codes that repeated throughout the 6 transcripts across clients and clinical staff.
- Stage 2: Codes were selected and would be used as the basis for coding in the next stage.
- Stage 3: Line-by-line coding was conducted, using the previously identified codes from stage 2 to guide reading and identifying analytically important text in the dialogue.
- Stage 4: Theoretical coding occurred, where all codes were considered collectively to generate conceptual categories (ie, themes).

## Results

### Overview

The in-depth qualitative focus group interviews were recorded on Zoom (Zoom Video Communications) and lasted up to 1 hour. As a result of the 6 focus group interviews (3 client groups and 3 staff groups of 4 to 8 individuals per group), Zoom-recorded audio material of 4 hours 40 minutes was obtained, which was then subjected to transcription. Groups were labeled (ie, client groups 1, 2, and 3; clinician groups 1, 2, and 3) solely for organizational purposes and do not reflect hierarchy or analytic distinctions. The 6 interviews, 3 from clients and 3 from clinical staff, were rich in depth and content. Following iterative engagement with the data, it was identified that saturation was reached once no new themes emerged, and there was no need to conduct repeat interviews. Iterative engagement consisted of repeated transcript review, reflexive memoing, and ongoing refinement of themes as analysis progressed, in line with Braun and Clarke’s reflexive thematic analysis [22]. Saturation was achieved when the data no longer yielded new insights or properties relevant to the emerging theoretical categories, and all core themes were fully developed and defined conceptually [22].

The identified codes that emerged were considered of greatest analytical importance due to their salience across interviews and relevance to the research questions. Analytic priority was given to codes that captured meaningful clinical patterns, rather than just those that appeared most frequently. The identified codes were as follows: client engagement, friendship, rapport, acuity, ADLs, client diagnosis, and use of clinical skills. These codes created 3 themes: community (client engagement, friendship, and rapport), clinical presentation (acuity, ADLs, and client diagnosis), and use of clinical skills.

### Theme 1: Community

#### Overview

The theme of community reflects the depth of connection fostered in in-person versus virtual mediums of mental health care. Across client and clinician groups, participants noted that while virtual formats provided continuity and flexibility, they also introduced barriers to interpersonal relating and opportunities for spontaneous interaction, all of which were described as essential to a sense of therapeutic community. For example, one clinician said:

*We’ve gotten to our ideal ... in person, no mask. We can see it all, and we have 100% of the information available to us in that moment ... it’s ... just—was the last barrier down in terms of like physical body language and stuff.*

[Staff group 1]

In this theme, community is explored thoroughly to fully saturate the concept through 3 subthemes: client engagement, friendship, and rapport. This section will explore how virtual care can serve as a valuable supplement to treatment, and where it may lack the relational qualities that contribute to the community, connection, and accountability of which in-person care naturally provides.

#### Client Engagement

Client engagement captured participants’ observations about attention, presence, and participation, particularly in group settings. Many described virtual sessions as easier to get distracted from or tune out of, especially when cameras were off or notifications were popping up on the client’s screen. If notifications are not manually turned off, it is understood that notifications may be distracting or cause the desire to “impulsively” check notifications instead of focusing on the session. Clinicians added that it is harder to address this lack of engagement when on Zoom (Textbox 1).

Textbox 1. **Lack of engagement.**

“It’s a lot easier to be distracted. Just by being in a different space, you have different things around you that can go off. Your phone can go off. If you’re on your computer, unless you make a conscious decision to turn off your notifications, you’re getting notifications. Versus if you’re in a group, it’s like you just put your phone away and if it buzzes you feel awkward because it’s buzzing and someone is talking ... And it’s more of a conscious decision to not be distracted on a computer screen than it is to be in group” [Client group 3].

“I found that in virtual sessions, while they’re still useful, there’s inevitably going to be implicit distractions. Even if you’re not explicitly trying to surf the web, you see your notifications everywhere. There’s a top clock in the corner of your screen. And impulsively sometimes I feel like I just click my email, and I go back but I think that takes away from the interpersonal experience and it allows me to talk about things at a superficial level where maybe the therapist wouldn’t notice” [Client group 1].

“I think that on Zoom or virtually, it’s harder to address engagement or lack of engagement, so that’s ... where it becomes a little different because ... it’s very easy for someone to shut off their video even though we ask them not to. But it’s hard for us to sort of pull them back out, whereas in person, it’s a lot easier to ask them a question and look them in the eye ... and sort of feel ... the group pressure or groupthink process to get them involved. But ... I think engagement is a little bit less on—virtually ‘cause it is easy to just sort of check out, look at other things” [Staff group 2].

Conversely, in-person groups were described as more immersive, often due to innate social pressure in being physically seen, subtle nonverbal cues, or eye contact that naturally invite participation, learning, and connection.

*When you’re in breakout rooms on Zoom, one, you’re probably in a room alone because you’re on Zoom, so you yearn for that social connection and you do that by talking about other things whereas in-person, you have time after group to talk about other things. I’m ... more focused on the subject matter of therapy with in-person groups.*

[Client group 1]

*I think it is largely more effective to be in person like everyone else has said too, but to have the flexibility and option can be nice and maybe encourage more participation if someone doesn’t feel like, “Oh, I have to be in person no matter what even if X, Y, Z comes up.”*

[Staff group 1]

#### Friendship

Friendship highlighted the organic ways that peer relationships tend to form before and after in-person sessions, like “water-cooler” talk. Participants emphasized that between-group sessions is where true connection and trust were built, a dynamic not easily replicated over Zoom, where interaction typically ends the moment the session does. For example:

*Definitely, proximity is huge in making friends and making connections with others. I’ve always found the people that I’m closest with are the people that I find myself in really close proximity with and being really close involves having a shared space like [peer] said. That’s a very helpful factor in making strong connections with others.*

[Client group 3]

*I mean, we have the clubhouse here and ... even if it’s not anything super clinical, like the ability after group to like all go down to the clubhouse, play ping-pong, play pool, go get some lunch. That definitely wasn’t happening virtually, and so I think it was definitely probably a lot harder for the clients to kinda connect with one another over Zoom, especially if they didn’t already know each other.*

[Staff group 1]

#### Rapport

Rapport focused on the alliance between clients and clinicians. While some participants described ways in which virtual care could support connection, such as learning clues about someone based on the way they decorate their home environment, many felt that unspoken exchanges and small talk were more organically cultivated in person. Additionally, a client shared that connection and community in person greatly reduced insecurities, leading to better engagement in treatment (Textbox 2).

Textbox 2. **Nonverbal communication and connection.**

“... let’s say we’re doin’ like an online DBT or somethin’ like that and we’re going through stuff. It was more of giving someone support with what they said instead of something like—it seemed very clinical. It didn’t seem organic” [Staff group 1].

“If I’ve heard somebody say something that I resonate with on a Zoom call, for example, I’ll make a point to ... reflect on that ... and really giving them a verbal hint that I’m understanding what they’re saying and nodding my head. Versus in-person, it’s easier to see body language and better response than virtually whereas you would verbally need to say it instead of in-person cues” [Client group 3].

“... being able to relate to people on a personal level and getting details about them that I would not otherwise know profoundly reduced my insecurities. When I came here, I was really insecure about the fact that I was a student on a leave of absence and I talked to people and they’re like, yeah, I’m also on a leave. And I’m sure maybe that would have came up but there’s just details and stuff you learn about people that you just wouldn’t know otherwise. And I think that’s really important in building a foundation so when you get to groups [where] you’re comfortable sharing, you feel better about your treatment” [Client group 1].

Some emphasized that the absence of shared physical space could subtly disrupt the therapeutic experience, due to rapport being lost. The concern is that this makes it harder to build trust and emotional connection. Some examples include:

*I think that there’s also the ability to pick up on ... body language and cues ... we’ll have clients who are anxious. Like their leg is bouncing or they’re fidgeting, and there’s things that you can pick up that you can’t pick up on virtually. And I also think having a space separate from their home and where they are allows for a different level of openness, and I think it can be hard to kind of really jump into the rapport-building in the same way virtually ...*

[Staff group 1]

*The disconnect comes from a place of personal experience with picking up on body language. And really connecting with others has a lot, for me, to do with experiencing others in their fullest sense and gaining a full experience of what others are saying versus on Zoom when we’re virtual and there’s not a full opportunity to gain the full experience with interacting with others. And granted, I’m definitely an extrovert, so I gain energy from being with others.*

[Client group 3]

### Theme 2: Clinical Presentation

#### Overview

Researchers also looked at clinical presentation, particularly as it relates to how a client shows up diagnostically (ie, how their symptoms are manifesting within the context of individual and group sessions and in virtual and in-person spaces). Clinical presentation was explored through 3 codes: client acuity, ADLs, and client diagnosis.

#### Acuity

When exploring client diagnostic acuity, openness, and engagement in individual and group therapy sessions across both mediums of care were discussed. Some staff felt that for clients in early recovery or in more of an acute psychiatric state, virtual sessions made it easier for clients to avoid addressing therapeutic issues head-on and even potentially engage in harmful behaviors behind the screen. Similarly, it was sometimes more difficult for clinicians to pick up on these behaviors virtually, decreasing the effectiveness of the therapeutic process. For example:

*If they’re in ... an environment that typically reinforces substance use or unhealthy behaviors ... I have heard stories about clients who were drinking ... alcohol out of a water bottle during sessions, and those sort of behaviors are obviously more difficult to do in an in-person setting. I think the high-acuity substance use also like takes a big hit when it’s virtual as opposed to in person*.

[Staff group 1]

*Yeah, I think similarly with eating disorders as well, any with high acuity, very similar to substance use ...*

[Staff group 1]

While some clinicians believed that in-person sessions were more effective for clients who needed higher levels of care and more oversight due to the severity of their symptoms, other clinicians also noted that virtual sessions could be a good option for those who are more stable (ie, in a maintenance stage of treatment, able to cope more effectively, and need less clinical oversight). For example:

*... if a client was then going back to college and ... wanting to be part of [mental health treatment] at like [outpatient level] it makes it a lot more accessible for them to be at [mental health treatment] if it’s virtual if their university is far away, but if a client were to come straight from [residential treatment program] or a hospital setting, whatever it is ... we would probably like ... to work with them more in person to get that important work done and then—until they maybe are a little bit more stabilized. Then we can kind of transfer it over to virtual*.

[Staff group 1]

*I think that in-person therapy is absolutely imperative, especially with groups. But I can see the functionality and use of Zoom therapy as a useful supplement or tool for busy young adults.*

[Client group 1]

#### Activities of Daily Living

ADLs explored a client’s ability to take care of their mental, physical, and emotional needs. Some clinicians felt that it was more difficult to pick up on how a client was taking care of their hygiene or physical health during virtual sessions, whereas in person, there may be more obvious signs if a person did not take good care of themselves (ie, whether a person showered, shaved, and took their medication).

*Even things like your ADLs. You can just walk past a client and smell whether or not this person took a shower or whatever. You could just see in a snapshot if they had a long night or whatever the situation is just walking around the place, seeing how they either get somethin’ to eat, how they socialize with other people.*

[Staff group 1]

*I think some of the thought disorders. I think without having certain clients come in person, there are certain hygiene factors that you ... you don’t know ... like when I have a client ... I can see other than just facial hair or their hair if there is—see or smell if there’s different hygiene. It tells me like more about if they’re taking their medication, how are they in their living, are they likely leaving their apartment at all or not, things like that.*

[Staff group 1]

Clients and staff felt that virtual sessions enabled clients to engage in avoidant behaviors, such as not needing to engage in ADLs like getting out of bed, getting dressed, showering, or taking medication. This avoidant behavior sometimes even made it difficult for clients to transition into things like jobs or other settings that were not virtual.

*You don’t have to get dressed, put shoes on, go outside, take the train, all that stuff, think about transit time.*

[Client group 1]

*... being at an intensive like IOP level of care, kind of like what [therapist]’s—, like the getting up and getting to a place is part of the treatment. It’s—a lot of people who haven’t been leaving their house or haven’t—whether it’s anxiety based, depression based, executive functioning ... I think even just getting up and getting dressed and the basic kind of behavioral activation things that that help with mood and anxiety are taken away in a virtual setting*.

[Staff group 1]

#### Client Diagnosis

When looking at diagnosis, researchers explored whether there were certain mental health diagnoses that were easier or harder to treat in a virtual session. Specifically, clinicians felt that virtual care was less effective for disorders like depression, agoraphobia, or social anxiety, where avoidance is prevalent, as it allowed clients to continue to stay in their homes and hide behind a screen, instead of addressing the issue more directly.

*I feel like it’s harder virtually to treat anyone. But I’m thinking of specifically people who like to avoid ... things, so maybe that’s because of anxiety or depression. But a lot of clients here wanna avoid, and I’ve had lots of clients who were like, “Can we meet virtual?” because they wanna avoid taking transportation, they wanna void coming to [mental health treatment] ... but part of doing those things is like facing your anxiety head on, so by keeping things virtual for some people, that keeps them in their avoidance zone*.

[Staff group 2]

*Social anxiety. Anxiety in general. For some people, part of the therapeutic process is leaving their place to go to another office, and that obviously takes out a big part of it. I had a client who would say like, “Oh, I can’t make it in today. I’m gonna be virtual,” when they were hybrid, and they definitely used it as avoidance. I think that with clients who one of their goals is to be in like a more public space and interacting, virtual therapy like takes out a huge chunk of that. And I think that ... anxious and socially anxious conditions ... like virtual really is incompatible with it*.

[Staff group 1]

Clinicians also felt that mental health conditions such as attention-deficit/hyperactivity disorder or thought disorders were often times more difficult to treat due to the amount of stimuli in the background and the potential for distractions.

*What comes to mind is folks with executive functioning disorders, attention deficit disorder, and folks with psychotic disorders are distractible from their own internal stimuli and don’t seem to do well with virtual*.

[Staff group 2]

*I feel like Attention Deficit Hyperactivity Disorder (ADHD), too, is another one because ... instead of you being in like a curated environment, you can be wherever. You can like walk around and someone might be in the other room, or ... there’s just so ... much room for more distractions*.

[Staff group 1]

Some clinicians also believe that eating disorders and substance use disorders are more difficult to treat virtually due to the lack of oversight in a virtual space. Specifically, due to the secretive nature and maladaptive behaviors of both disorders, it is harder to assess whether clients are using substances, purging, or even eating their meals through a screen (Textbox 3).

Textbox 3. **Visibility and accountability.**

“I’ve also had a couple clients with like body image issues who didn’t like to see their face on screen, and they’d hide behind like ... a ... photograph of a dog and just like would not engage and ... it was kind of disruptive in the group. I understood where they were coming from, but it was hard to find a workaround for that” [Staff group 1].

“I think it’s impossible to do a truly beneficial EDO meal group virtually as opposed to in person just because of the amount of ED behaviors that would even be an option for the clients. Like I can imagine that like just having that sort of opportunity to engage in these behaviors would be either tempting or distressing to those in the group.I think that ... a successful virtual [eating disorders] meal group would be next to impossible” [Staff group 1].

“I think being in person and like seeing someone walk and sit down and being with them face to face is really helpful when you’re dealing with folks who use substances. So there’s just ... an added ... ability to, I think, go unnoticed and for folks to evade detection or toxicologies or any sort ... anything that will like get them found out. I think that’s probably similar with eating disorders. We’ve seen people just completely refuse to like show how much they’ve eaten, or they just deny that they like went to the restroom right after eating” [Staff group 3].

There were some disorders, however, where clinicians and clients both thought that virtual sessions or groups could be appropriate and helpful depending on things like client age and the content of the therapy group, sometimes making it easier for clients to use coping skills in a comfortable space after an intense group.

*My adolescent client ... we can do really, really good work, but due to her anxiety, like she is able to do makeup and like work on her hair and stuff like in session, which ... it also feels like very age appropriate, and I know she gets like super fidgety with her anxiety*.

[Staff group 3]

*... in terms of groups that are entirely virtual, the participation does depend group to group but there have been times where especially at trauma groups it’s helpful to be virtual. I think there’s less of a sense of vulnerability and I realize ... the goal is to be comfortable with vulnerability. But especially early on in treatment being in trauma groups ... it was helpful because I could sit on my bed and I could do my little things that help me calm down and I didn’t feel like everyone was seeing me even though I was on camera, so in that way it was a lot more helpful.*

[Client group 2]

### Theme 3: Use of Clinical Skills

Finally, researchers looked at the use of their clinical skills and how the medium of care (ie, virtual vs in-person) impacts this, if at all, within the context of individual and group sessions. Use of clinical skills was both the theme and code for this category, with particular topics coming up around the importance of engagement, rapport building, and body language being a cornerstone for using clinical skills.

For example, engaging clients is a skillset that clinicians highlighted as difficult when comparing virtual and in-person therapeutic spaces. Clinicians reported that client engagement skills are negatively impacted in virtual spaces:

*It was a deep, deep challenge to have the clients reflect until you ... found that sweet spot ... But if it was left up to, “Okay, you guys, what did you wanna talk about?” dead silent. And then working through just the different personalities ... so I didn’t know where my leverage was with them or what I could use to engage them.*

[Client group 3]

Fostering therapeutic alliance and rapport is another clinical skill that appeared in the transcripts when discussing the use of virtual versus in-person therapeutic spaces. Clinicians found that rapport building was easier in person. For example:

*A big way I develop rapport is through humor, and I found that it’s harder to be humorous or to exude my type of humor on Zoom ‘cause just you get less ... bodily feedback and body language confirmation, so it can make me proceed a little bit more shyly on Zoom ... it’s definitely about ... making sure to take that time and not like speed through something just because you have their attention and you’re face to face with them.*

[Staff group 2]

Clients and clinicians also noted that picking up on body language cues, an important domain of identifying signs and symptoms of mental health concerns, is more challenging in a virtual space. Being able to only see one’s head prevents the clinician from accessing important nonverbal cues (ie, tapping fingers and legs shaking; Textbox 4).

Textbox 4. **Challenges in a virtual space.**

“I think it’s just as difficult for [clinicians] to understand and realize my mannerisms ... I have a very difficult time, so I think anybody else would have a difficult time as well” [Client group 3].

“... all you can see is the square of their head. You can’t see what they’re doing with their legs, with their hands, who’s on each side of them. It’s not a real representation of therapy” [Staff group 2].

“I was trained as a psychoanalyst and group psychotherapist so seeing and lookin’ at people’s body language or a facial expression ... You have to really do a extra tune-in to someone that’s virtual to see because again if it’s just chest and above, you’re not seein’ other things. Is their leg movin’ rapidly? Are they tappin’ their fingers? Just you can’t see as much as if they were in person, so it does take a shift in the way you read the room or read all group members, so there is that limitation there” [Staff group 2].

Finally, eye movement desensitization and reprocessing (EMDR) was mentioned multiple times as a modality that clinicians felt strongly about being conducted in person. The below quote summarizes the consensus:

*I personally would prefer Eye Movement Desensitization and Reprocessing (EMDR) in person ... because it is so intense and because so much stuff can come up for a client, it’s important to have a safety zone of being in an office with a clinician. And you really, really need to be able to read your clients during EMDR.*

[Staff group 2]

## Discussion

### Principal Findings

While virtual care is acknowledged by the study participants as a valuable tool for maintaining treatment continuity, particularly in the face of logistical barriers (ie, distance, scheduling, school, or work), participants repeatedly emphasized the relational and clinical limitations innate to virtual platforms. This study uncovers, from the perspective of client and clinician focus groups, that in-person treatment is more supportive and fosters engagement in treatment, interpersonal connection (ie, therapeutic alliance and peer-to-peer), and the ability to exercise clinical skills than virtual settings.

Clinicians reported that their ability to use essential clinical skills (ie, engaging clients, fostering therapeutic alliance, and reading body language) is hindered in virtual settings. Certain modalities, such as EMDR, were considered more effective in person due to the emotional intensity they evoke and the importance of clinicians closely monitoring clients for safety in this type of therapeutic modality. While existing literature supports the feasibility of EMDR in virtual settings, there is a need for more robust methodological research to examine efficacy [22]. This study supports in-person treatment as the ideal medium for complex modalities, such as EMDR, and high acuity cases of all diagnoses, given the emotional and relational nuance that is missing when treatment is conducted online.

In examining how therapeutic relationships and community dynamics differ across virtual and in-person care, it was found that loss of connection occurs when therapy switches to a virtual format. Clients described distractibility and a reduction in therapeutic and peer-to-peer accountability when in virtual groups. Body language, nonverbal communications, and water-cooler conversations between sessions were some of the factors that were reported as being essential for therapeutic alliance and peer connection; yet, these were also reported to mainly occur in an in-person setting. Participants’ reports made clear that there are qualities of in-person care that help build rapport and foster therapeutic alliance, naturally, in a way that only virtual settings can forcibly achieve. Existing literature supports the finding that virtual mental health treatment limits access to nonverbal cues, body language, and the type of connection that is central to therapeutic and peer alliance [5,17,24].

Acuity of mental health diagnoses also strongly influenced participant perceptions of the medium of care. When discussing clinical presentation, clinicians and clients admitted to the absence of and difficulty in noticing subtle cues (eg, smell and fidgeting) that are essential for picking up on signs and symptoms of mental health concerns. For this reason, in-person care was reported as a necessity for clients with higher acuity mental health concerns and complex diagnoses, such as active eating, substance use, or thought disorders.

### Implications

This study extends previous work that examines the benefits and drawbacks of virtual mental health care [5]. The ability to connect, be present, and experience therapeutic alliance as well as support, particularly for young adults, is understood to be most effective when in person. While each setting offers distinct advantages, participants emphasized that clients with higher clinical acuity and complex diagnoses experience more effective support, safety, and peer-to-peer as well as therapist-to-client connection when in person. Certain diagnoses may need more support and the added element of in-person monitoring for safety, due to the complexity and/or acuity of one’s mental health condition. These findings underscore the importance of accounting for therapeutic and diagnostic factors when deciding the medium of care, as this can offer an effective framework for optimizing client engagement and experience.

### Limitations

The following limitations are consistent with Cerrito et al [5]. In summary, these include the following: (1) the analyzed group is not representative of the broader populations of mental health providers and young adults seeking mental health treatment, and future research should examine these hypotheses in a larger, more diverse population. (2) Considering factors that qualify a client for virtual versus in-person care is warranted in future research, as we understand that these results represent a specific group of individuals at a particular point in history (ie, post–COVID-19 pandemic), from large urban settings (ie, New York, NY, and Washington, DC), and social contexts (ie, predominantly high socioeconomic status). (3) Due to the necessary exclusion criteria, the sample may not fully represent all vulnerable populations. Individuals were excluded if they were unable to complete the survey due to medical or psychological limitations or if participation required a legally authorized representative.

### Conclusions

The results of this study outline how the medium of care (ie, virtual vs in- person) impacts therapeutic experience from the perspective of both client and clinician. It is understood that critical domains such as community, interpersonal relationships, and clinicians’ use of clinical skills are affected by the medium of care, and key factors such as clinical presentation (ie, acuity and diagnosis) must be accounted for when choosing a particular mode of care for a client.

## Abbreviations

ADL: activities of daily living
EMDR: eye movement desensitization and reprocessing
